# Pantera Lux Drug-Coated Balloon for the Treatment of Coronary Artery Lesions in Routine Practice

**DOI:** 10.3390/jcm14093133

**Published:** 2025-05-01

**Authors:** Rayyan Hemetsberger, Nader Mankerious, Kevin Hamzaraj, Ahmed Alali, Gert Richardt, Ralph Tölg

**Affiliations:** 1 Department of Cardiology, Internal Medicine II, Medical University of Vienna, 1090 Wien, Austria; 2 Heart Center, Segeberger Kliniken, 23795 Bad Segeberg, Germany; 3 Department of Cardiology, Zagazig University, Zagazig 44519, Egypt; 4 Asklepios Clinic, 23843 Bad Oldesloe, Germany; 5 Medical Faculty of the Christian-Albrechts, University of Kiel, 24118 Kiel, Germany

**Keywords:** drug-coated balloon, paclitaxel, Pantera Lux

## Abstract

**Background/Objectives:** We sought to confirm the performance and safety of the Pantera Lux paclitaxel-coated balloon (pDCB) when used as per the instructions for use at a single high-volume center. **Methods:** In this retrospective analysis, 386 consecutive patients were categorized into three groups: the treatment of drug-eluting stent in-stent restenosis (DES-ISR) lesions (n = 191), bare-metal stent in-stent restenosis (BMS-ISR) lesions (n = 127), and de novo lesions (n = 68). The primary endpoint at 12 months was target-lesion revascularization (TLR). Secondary endpoints were device success, target-vessel myocardial infarction (TV-MI), and cardiac death. **Results:** The baseline characteristics were balanced between the groups, with a median age of 71.3 years, 25% being female, 32% being diabetic. The majority presented with chronic coronary syndrome (82.9%). Type C lesions were more often observed in the DES-IRS group as compared with the BMS-IRS and de novo groups (15.6% vs. 7.9% vs. 7.4%, *p* < 0.001). Cutting balloons were more often used in the DES-IRS group (41.0% vs. 19.7% vs. 1.5%, *p* < 0.001). The residual stenosis rate was 7.6% vs. 3.3% vs. 7.3% (*p* = 0.002). The TLR at 12 months was 8.9% vs. 2.4% vs. 1.5% (*p* = 0.013). Device success was achieved in 98.8% vs. 98.5% vs. 100% of cases (*p* = 0.8). TV-MI occurred in 3.2% vs. 0.8% vs. 1.5% (*p* = 0.5) and cardiac death in 2.6% vs. 0.0% vs. 2.9% (*p* = 0.13) in DES-IRS vs. BMS-IRS vs. de novo lesions. **Conclusions:** In this single-center observation, we confirmed the safety and efficacy of the Pantera Lux paclitaxel-coated balloon for the treatment of DES-IRS, BMS-IRS, and de novo lesions with low TLR rates at 12 months.

## 1. Introduction

Drug-coated balloons (DCBs) were introduced to clinical practice around two decades ago for the treatment of coronary artery lesions and have since been available in Europe [1]. Initially, the clinical use of paclitaxel-coated balloons (PCBs) focused on in-stent restenosis (ISR) and the treatment of small coronary arteries. However, with the advent of the newer generation drug-eluting stents featuring more biocompatible coatings, DCBs—with their paclitaxel coatings—have lost importance in current guidelines [2]. Nevertheless, with challenges related to coating balloons with sirolimus having been overcome, interest in DCB technology, including older paclitaxel-coated DCBs, is renewed [3].

Following the release of the AGENT IDE randomized trial data, the first DCB has obtained FDA approval for treating coronary lesions [4]. As of today, there are two antiproliferative drugs—paclitaxel and -limus derivates—that are applied in different formulations to improve the process of drug release and tissue uptake [5]. The goal of this local antiproliferative drug application after successful lesion preparation is to limit neointimal formation, translating to reduced late lumen loss (LLL) and reduced need for repeated revascularization [6,7]. Nevertheless, the clinical outcome of a DCB treatment strategy is not only dependent on the antiproliferative drugs used but also on adequate and meticulous lesion preparation prior to the DCB treatment [8]. ISR lesions present mostly in larger proximal vessels and are more difficult to prepare, while de novo small-vessel disease can be found mainly in distal vessels or side branches and requires careful preparation, as bail-out stenting would cause higher restenosis rates [9].

Considering this renaissance of DCBs, we sought to investigate the clinical performance of the paclitaxel-coated balloon Pantera Lux (Biotronik, Bülach, Switzerland) in real-world clinical practice.

## 2. Materials and Methods

### 2.1. Study Design and Study Population

This is a retrospective analysis of patients treated with the Pantera Lux DCB at a single high-volume center, the Heart Center Bad Segeberg, Germany. Patients with a complete follow-up period of one year were included during the 2022–2023 period based on the following treatment indications: DES in-stent restenosis lesions (n = 191), BMS in-stent restenosis lesions (n = 127), and de novo lesions (n = 68). The decision for use of DCB was left to the discretion of the operator.

### 2.2. Study Device

The Pantera Lux paclitaxel-coated balloon received CE marking on 22 September 2009. The balloon’s surface is homogeneously coated with a delivery matrix containing 3 μg of paclitaxel per mm^2^. Paclitaxel is delivered to the vessel wall upon balloon expansion. A balloon inflation time of 30 s should be adequate for sufficient drug transfer. The available balloon diameters range from 2.0 mm to 4.0 mm in 0.5 mm increments, and the balloon lengths range from 10 mm to 30 mm in 5 mm increments. A short list of settings in the instructions for use is attached in the Supplementary Materials.

### 2.3. Endpoint Definition

The primary endpoint is clinically driven target-lesion revascularization (cd-TLR) at 12 months post-index procedure. The primary endpoint was analyzed per patient and only the first TLR event was considered. TLR was defined in accordance with the ARC-2 definitions [10], with documentation of recurrent clinical symptoms of the patient and ≥50% diameter stenosis at the target lesion or a diameter stenosis of ≥70%, regardless of the presence or absence of ischemic signs or symptoms. The target lesions of in-stent stenosis involved the adjacent proximal and distal 5 mm segments [10]. As a secondary endpoint, device success was evaluated for all Pantera Lux balloon catheters for which the foil pouch had been opened. Device success was defined as Pantera Lux delivery to the target-lesion site, appropriate balloon inflation and deflation, and removal of the delivery system. Device success was evaluated through the index procedure. Further secondary endpoints were cd-TLR, target-vessel myocardial infarction (TV-MI), and cardiac death at one, six and twelve months. TV-MI was defined as a myocardial infarction involving the target-vessel territory, following the fourth universal definition of MI [11]. Cardiac death was defined as per the ARC-2 definitions [10].

Hospital records were thoroughly screened by a certified study coordinator for any suspected endpoint event. Follow-up was performed by certified study coordinators by phone contact using standardized questionnaires. In case of any rehospitalizations or repeated coronary angiography, the respective medical documentation was acquired to screen for endpoint events. Every endpoint event was reviewed by an investigator.

### 2.4. Statistical Analysis

The sample size calculation was based on the primary endpoint. To calculate the sample size for the DES-ISR, BMS-ISR, and de novo lesion cohorts, a precision-based calculation approach was chosen using the exact Clopper–Pearson 95% confidence interval (CI) method. A detailed description is attached in the Supplementary Materials (Supplementary Tables S1–S6). Categorical variables are presented as frequencies and percentages, while continuous variables are summarized as mean ± SD or median [25th–75th quartiles], depending on variable distribution. Inter-group comparisons were conducted using Fisher’s exact test and Pearson’s chi-squared test for categorical variables, and the Kruskal–Wallis test for continuous variables. Clinical endpoints were reported as crude rates at 12 months and compared using Fisher’s exact test. Binary logistic univariable analyses were performed for the patient-level variables with the occurrence of a target-lesion revascularization at 12 months as the binary outcome. When separation occurred, the Firth logistic regression was performed. Variables with *p*-values < 0.05 were entered into the multivariable analysis. The analyses were performed using R, version 4.3.3. A *p*-value of <0.05 was considered the level of statistical significance.

## 3. Results

For this analysis, a total of 386 patients were included. Of them, 49.5% (n = 191) were treated with a Pantera Lux for DES-ISR, 32.9% (n = 127) for BMS-ISR, and 17.6% (n = 68) for de novo lesions.

### 3.1. Baseline Characteristics

The baseline characteristics are listed in Table 1. The mean age was 71.3 years, 25% were female, and 32% were diabetic, with no significant differences between groups. Patients with ISR were more often hyperlipidemic than patients with de novo lesions. The majority of patients presented with chronic coronary syndrome (82.9%), while 1.6% presented with STEMI, 8.8% with NSTEMI, and 6.7% with unstable angina.

### 3.2. Lesion and Procedural Characteristics

Lesion characteristics are summarized in Table 2. Bifurcation lesions were more prevalent in the de novo lesion group, followed by the DES-ISR group and BMS-IRS groups (33.8% vs. 27.8% vs. 11.8%, *p* < 0.001). Type C lesions were more often observed in the DES-IRS group as compared with the BMS-IRS and de novo groups (15.6% vs. 7.9% vs. 7.4%, *p* < 0.001). The reference vessel diameters were smaller in de novo lesions than in ISR lesions (3.1 ± 0.5 mm vs. 3.0 ± 0.4 mm vs. 2.1 ± 0.3 mm, *p* < 0.001 for DES-ISR vs. BMS-ISR vs. de novo lesions). Diameter stenosis was 83.3% for DES-ISR, 80.4% for BMS-ISR, and 88.3% for de novo lesions (*p* < 0.001).

Procedural characteristics are listed in Table 2. The predominant lesion preparation technique was plain balloon angioplasty (80.8%) followed by cutting balloons (27.8%) and scoring balloons (14.0%). Plain balloon angioplasty was used in 77.4% of DES-ISR cases, 80.3% of BMS-ISR cases, and 92.6% of de novo lesion cases (*p* < 0.001), whereas cutting balloons were more often used in the DES-ISR group than in the BMS-ISR and de novo groups (41.0% vs. 19.7% vs. 1.5%, *p* < 0.001). Pantera Lux balloon diameters were similar in the DES-ISR and BMS-ISR groups but smaller in the de novo lesion group (3.1 ± 0.5 mm vs. 3.0 ± 0.4 mm vs. 2.1 ± 0.3 mm, *p* < 0.001). Residual stenosis was 7.6% vs. 3.3% vs. 7.3% for DES-ISR vs. BMS-ISR vs. de novo (*p* = 0.002).

### 3.3. Clinical Endpoints

**Figure 1:**
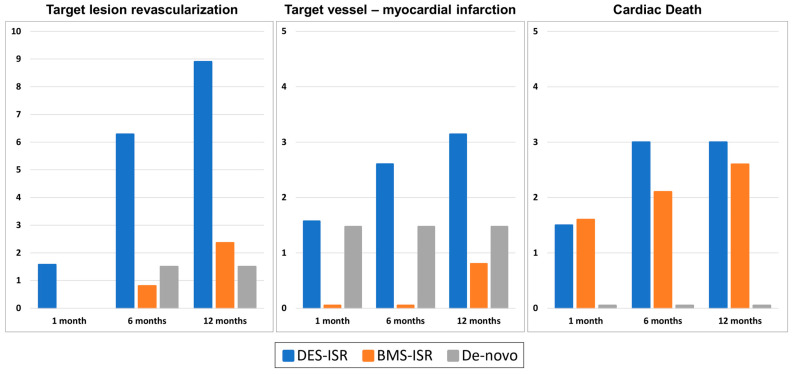
Clinical endpoints.

Clinical endpoints are listed in Table 3 and depicted in Figure 1. Device success was achieved in 98.8% vs. 98.5% vs. 100% of cases (for DES-ISR vs. BMS-ISR vs. de novo, *p* = 0.8). The crude rate of cd-TLR at 12 months was 8.9% vs. 2.4% vs. 1.5% for DES-ISR vs. BMS-ISR vs. de novo (*p* = 0.027). After accounting for potential confounders, DES-ISR emerged as an independent predictor for TLR at 12 months (OR 4.67 [95%CI: 1.73–15.46], *p* = 0.002) (Supplementary Table S7). Within one year, TV-MI occurred in 3.2% vs. 0.8% vs. 1.5% (*p* = 0.5) and cardiac death in 2.6% vs. 0.0% vs. 2.9% (*p* = 0.13) in DES-ISR vs. BMS-ISR vs. de novo lesions. Further results of secondary endpoints at one and six months are demonstrated in Figure 1.

## 4. Discussion

In this retrospective analysis, clinical event rates following treatment with the Pantera Lux PCB were low for both ISR and de novo coronary lesions. TLR rates were higher in cases of DES-ISR compared to BMS-ISR or de novo lesions following PCB treatment. However, rates of device success, TV-MI, and cardiac death at 12-month follow-up were not statistically different across all lesion types.

The theoretical advantage of using DCBs lies in avoiding the implantation of a permanent metallic cage within the vessel, thereby mitigating inflammation-driven ISR and reducing the risk of thrombosis. DCBs can be applied across a wide range of clinical scenarios and lesion subsets. In patients at high bleeding risk, the potential to shorten the duration of DAPT is particularly beneficial. Complex interventions, such as those involving bifurcation lesions, may be simplified by using a DCB to deliver the antiproliferative agent to the vessel wall after adequate lesion preparation, thereby avoiding the need for complex two-stent strategies. Similarly, in CTO interventions, where distal runoff may involve hypoplastic vessels, DCBs offer a viable alternative. The same holds true for diffusely diseased long lesions lacking healthy landing zones for DES implantation, as well as for small vessel disease, where DCBs are frequently favored over DES. In summary, DCBs can be used in ISR to prevent the accumulation of multiple metal layers, as well as across a variety of de novo lesion subsets [12].

In recent years, interventional cardiology has seen renewed interest in DCBs. Stents were originally developed as bailout devices to treat flow-limiting dissections after plain balloon angioplasty. DESs were subsequently introduced to address the high ISR rates observed with BMS. Among early DESs, sirolimus demonstrated superior efficacy and safety over paclitaxel, owing to its cytostatic mechanism of action and broader therapeutic window, while paclitaxel is cytotoxic with a narrower therapeutic range [13]. As a result, newer-generation DESs, all eluting -limus drugs, have achieved low clinical event rates, which contributed to a decline in the use of paclitaxel-based DCBs. However, the development of stable sirolimus-coating technologies for balloons has led to renewed interest in DCBs, and both paclitaxel- and sirolimus-coated balloons are now being utilized in the aforementioned lesion and patient subsets.

Although drug-coated balloons are being increasingly adopted in clinical practice, the extent and generalizability of the supporting evidence are still being discussed. While several studies suggest a clinical benefit in selected indications, others have limitations, including small sample sizes, limited statistical power, and a focus on specific lesion subtypes. As a result, current guideline recommendations remain cautious, despite growing real-world experience. European and U.S. guidelines [2,14] currently do not mention DCB use in de novo lesions, and the ESC guidelines [2] recommend DESs over DCBs for the treatment of DES-ISR. This recommendation is based on data from the long-term follow-up of the ISAR-DESIRE 3 trial [15], the DAEDALUS individual patient-level meta-analysis [16], and another trial-level meta-analysis [17]. However, these recommendations have drawn criticism. The referenced studies did not employ standardized lesion preparation protocols and were not guided by intracoronary imaging, and in the DAEDALUS analysis, all-cause mortality was numerically higher in the DES arm than in the DCB arm. Given the pivotal role of lesion preparation [8,12] and intracoronary imaging [18] in the treatment of ISR, many interventionalists continue to prefer DCB use over the implantation of additional DESs in ISR cases.

Meanwhile, additional data have emerged on the use of DCBs in de novo and bifurcation lesions. In the REC-CAGEFREE I trial [19], the paclitaxel-coated balloon (PCB) Swide^®^ (Shenqi Medical, Shanghai, China) failed to demonstrate non-inferiority to a sirolimus-eluting stent with respect to the primary composite endpoint of cardiovascular death, TV-MI, and TLR. Notably, subgroup analyses revealed no statistically significant differences in the primary endpoint between PCBs and DESs in patients with small-vessel disease and bifurcation lesions. The overall lesion complexity in the trial was low, with reported SYNTAX scores of 7.3 and 7.4. Given the ongoing refinement and excellent performance of contemporary DESs, it is conceivable that drug-coated balloons may demonstrate their greatest benefit in more complex anatomical subsets. In contrast, in relatively straightforward lesions—where DES outcomes are already optimized—larger differences are not necessarily expected. Importantly, the Swide^®^ PCB used in this trial is not approved for use in Europe or the United States, which limits the generalizability of the findings to these geographies. Additional evidence supporting the use of PCBs in small-vessel de novo disease stems from the ANDROMEDA meta-analysis [20], an individual patient-level pooled analysis of randomized controlled trials. PCB treatment resulted in a significant reduction in the primary endpoint (composite of cardiac death, TV-MI, TLR) compared to DESs, primarily driven by lower rates of TV-MI and TLR. Data on DCB use in the side branch of true bifurcation lesions were provided by the DCB-BIF study [21]. In this trial, DCB therapy demonstrated superiority over non-compliant balloon angioplasty for the treatment of the side branch following crossover stenting of the main branch, when side branch intervention was deemed necessary. The primary endpoint—a composite of cardiac death, TV-MI, and TLR—was significantly more frequent in the non-compliant balloon group, largely due to an increased incidence of TV-MI. The underlying mechanism for this finding remains unclear. Nonetheless, the body of evidence in favor of DCB use continues to grow. Results from the ongoing SELUTION DeNovo Trial [22], which investigates the sirolimus-eluting balloon Selution™ (Cordis, Miami, FL, USA) versus a DES in de novo coronary lesions, are anticipated at TCT 2025 and may further clarify the role of DCBs in contemporary practice.

In our study, we observed a high TLR rate of 8.9% at one-year follow-up in DES-ISR treated with the Pantera Lux PCB. Also, higher TLR rates have been reported in the BIOLUX trial (15.7%) [23], RIBS IV trial (16%) [24], and ISAR-DESIRE 3 trial (24%) [3]. In the most recent and largest randomized DCB trial to date, AGENT IDE [4], a paclitaxel-coated balloon (Agent PCB, Boston Scientific), was compared with an uncoated balloon in a population with 89% DES-ISR. The one-year TLR rate was 13% in the DCB group versus 24.7% in the control group.

Consistent with previous findings, our study also demonstrated lower TLR rates in BMS-ISR compared to DES-ISR. For instance, the BIOLUX trial [23] reported a TLR rate of 9.8% in BMS-ISR. Similarly, in the PEPCAD II trial [25], PCB treatment in BMS-ISR resulted in a TLR rate of 6.3%. The difference in TLR outcomes between BMS-ISR and DES-ISR is well established and primarily attributed to differences in the pathophysiological substrate of restenosis. BMS-ISR is predominantly characterized by smooth muscle cell-rich neointimal hyperplasia, while DES-ISR is associated with more complex and heterogeneous neoatherosclerotic changes [26].

In the subgroup of de novo lesions in small coronary arteries, we observed very low to almost absent TLR rates. Randomized trials have likewise reported TLR rates ranging from 3% to 6% [9,27,28]. The more stable clinical presentation in this patient group may have contributed to these favorable outcomes.

Paclitaxel appears to provide the effective suppression of neointimal proliferation across different lesion types. Nevertheless, the development of sirolimus-based DCBs using biocompatible formulations that may overcome previous pharmacological limitations is currently underway [29]. Sirolimus-coated DCBs may be particularly attractive to operators seeking to deliver the standard antiproliferative agent used in DESs without the need for permanent implants. However, the actual clinical impact of this strategy remains to be established, as current head-to-head comparisons have not yet demonstrated the superiority of -limus-based antiproliferative therapy over paclitaxel in the context of DCBs [29]. Importantly, DCBs must first demonstrate superiority or at least non-inferiority to current-generation DES in both de novo lesions and ISR within adequately powered RCTs. Only after such data become available will head-to-head comparisons between different DCB platforms be meaningful.

However, despite the potential clinical benefits of DCBs, there are significant challenges to their broader adoption in everyday practice. One major hurdle is the substantial cost disparity between DCBs and DESs. DCBs are considerably more expensive than current-generation DESs, placing an economic burden on patients and healthcare systems. This financial strain may limit the widespread use of DCBs, particularly in resource-constrained settings. In addition to the cost issue, successful implementation of a DCB strategy requires careful lesion preparation, which demands skill and experience from operators. Techniques such as the use of cutting balloons, starting lesion preparation with smaller balloons, and gradually increasing to a 1:1 balloon-to-vessel ratio are critical to optimizing DCB performance. Furthermore, operators must understand how to manage dissections and intramural hematomas, which are normally simply stented. These technical requirements necessitate specialized training and a learning curve, further complicating the adoption of DCB strategies in clinical practice.

### Limitations

Firstly, the presented evaluation is based on data derived from a single center. Second, a decision for the use of DCB for treatment was left at the discretion of the operator. This may have led to bias wherein, after lesion preparation, only more suitable lesions may have been treated using a DCB and more unfavorable results after predilation (e.g., residual stenosis or dissections) were finalized with a stent following the third report of the international consensus group [8]. However, due to this, the actual analysis reflects the real treatment scenario, where the operator decides on device use in the individual setting according to his experience, which may have influenced outcomes. Third, this analysis is based on registry data, and despite the thorough and standardized medical review and follow-up, an underreporting of events cannot be totally ruled out, thus leading to somewhat lower endpoint events than in prospective randomized trials. However, the chosen endpoints are not prone to underreporting.

## 5. Conclusions

Our study of a high-volume interventional center based on a real-world patient cohort with ISR or de novo small-vessel disease suggests that the use of Pantera Lux PCB is safe and exhibits good clinical performance at 12-month follow-up. DES-ISR showed increased rates of TLR compared to BMS-ISR or small-vessel treatment. The use of this DCB may warrant low clinical events in chronic coronary syndrome; however, future studies are needed to investigate the given implications in dedicated lesion types and clinical subgroups.

## Figures and Tables

**Table 1:** Baseline characteristics.

	Total Cohort (n = 386)	DES-ISR (n = 191)	BMS-ISR (n = 127)	De Novo (n = 68)	*p*-Value
Age	71.3 ± 9.7	71.5 ± 9.7	70.9 ± 9.8	71.5 ± 9.7	0.7
Female	96/386 (24.9%)	42/191 (22.0%)	33/127 (26.0%)	21/68 (30.9%)	0.3
BMI	28.0 ± 4.4	28.3 ± 4.8	28.0 ± 3.8	27.2 ± 4.6	0.4
Hyperlipidemia	244/386 (63.2%)	127/191 (66.5%)	85/127 (66.9%)	32/68 (47.1%)	0.010
Hypertension	365/386 (94.6%)	182/191 (95.3%)	121/127 (95.3%)	62/68 (91.2%)	0.4
Diabetes mellitus	123/386 (31.9%)	69/191 (36.1%)	31/127 (24.4%)	23/68 (33.8%)	0.4
Smoking history	162/386 (42.0)	98/191 (50.8%)	40/127 (31.5%)	25/68 (36.8%)	0.01
LVEF [%]	52.3 ± 11.4	51.0 ± 11.9	54.1 ± 10.8	52.5 ± 11.0	0.092
Previous PCI	363/386 (94.0%)	191/191 (100.0%)	127/127 (100.0%)	45/68 (66.2%)	<0.001
Peripheral artery disease	63/386 (16.3%)	38/191 (19.9%)	16/127 (12.6%)	9/68 (13.2%)	0.2
Carotid artery disease	84/386 (21.8%)	45/191 (23.6%)	28/127 (22.0%)	11/68 (16.2%)	0.4
Impaired renal function	67/386 (17.4%)	39/191 (20.4%)	20/127 (15.7%)	8/68 (11.8%)	0.4
Clinical presentation					0.854
STEMI	6/386 (1.6%)	3/191 (1.6%)	2/127 (1.6%)	1/68 (1.5%)	
NSTEMI	34/386 (8.8%)	17/191 (8.9%)	10/127 (7.9%)	7/68 (10.3%)	
Unstable angina	26/386 (6.7%)	18/191 (9.4%)	5/127 (3.9%)	3/68 (4.4%)	
Chronic coronary syndrome	320/386 (82.9%)	153/191 (80.1%)	110/127 (86.6%)	57/68 (83.8%)	

**Table 2:** Lesion and procedural characteristics.

	Total Cohort % (n = 386)	DES-ISR (n = 191)	BMS-ISR (n = 127)	De Novo (n = 68)	*p*-Value
LESION CHARACTERISTICS					
No. of lesions	407	212	127	68	
Target Vessel					<0.001
LM	8/407 (2.0%)	8/212 (3.8%)	0/127 (0.0%)	0/68 (0.0%)	
LAD	145/407 (35.6%)	68/212 (32.1%)	38/127 (29.9%)	39/68 (57.4%)	
LCX	100/407 (24.6%)	48/212 (22.6%)	31/127 (24.4%)	21/68 (30.9%)	
RCA	134/407 (32.9%)	74/212 (34.9%)	53/127 (41.7%)	7/68 (10.3%)	
Venous CABG	20/407 (4.9%)	14/212 (6.6%)	5/127 (3.9%)	1/68 (1.5%)	
ISR Mehran classification					0.6
Class I		103/212 (48.6%)	70/127 (55.1%)	-	
Class II		68/212 (32.1%)	34/127 (26.8%)	-	
Class III		20/212 (9.4%)	9/127 (7.1%)	-	
Class IV		21/212 (9.9%)	14/127 (11.0%)	-	
Lesion type AHA/ACC class					<0.001
Type A	37/407 (9.1%)	4/212 (1.9%)	17/127 (13.4%)	16/68 (23.5%)	
Type B1	212/407 (52.1%)	104/212 (49.1%)	70/127 (55.1%)	38/68 (55.9%)	
Type B2	110/407 (27.0%)	71/212 (33.5%)	30/127 (23.6%)	9/68 (13.2%)	
Type C	48/407 (11.8%)	33/212 (15.6%)	10/127 (7.9%)	5/68 (7.4%)	
Bifurcation	97/407 (23.8%)	59/212 (27.8%)	15/127 (11.8%)	23/68 (33.8%)	<0.001
Calcification					<0.001
Moderate	146/407 (35.9%)	86/212 (40.6%)	54/127 (42.5%)	6/68 (8.8%)	
Severe	44/407 (10.8%)	39/212 (18.4%)	3/127 (2.4%)	2/68 (2.9%)	
Reference vessel diameter [mm]	2.9 ± 0.6	3.1 ± 0.5	3.0 ± 0.4	2.1 ± 0.3	<0.001
Lesion length [mm]	13.3 ± 9.6	14.6 ± 11.7	11.4 ± 6.3	12.6 ± 6.2	0.01
Diameter stenosis [%]	83.2 ± 11.3	83.3 ± 10.4	80.4 ± 12.8	88.3 ± 9.5	<0.001
PROCEDURAL CHARACTERISTICS					
Lesion preparation technique					
POBA	329/407 (80.8%)	164/212 (77.4%)	102/127 (80.3%)	63/68 (92.6%)	<0.001
Cutting	113/407 (27.8%)	87/212 (41.0%)	25/127 (19.7%)	1/68 (1.5%)	<0.001
Scoring	57/407 (14.0%)	28/212 (13.2%)	26/127 (20.5%)	3/68 (4.4%)	<0.001
Rotational atherectomy	2/407 (0.5%)	2/212 (0.9%)	0/127 (0.0%)	0/68 (0.0%)	0.006
Intravascular lithotripsy	1/407 (0.2%)	1/212 (0.5%)	0/127 (0.0%)	0/68 (0.0%)	0.005
Thrombus aspiration	1/407 (0.2%)	1/212 (0.5%)	0/127 (0.0%)	0/68 (0.0%)	0.005
Pantera Lux Balloon					
No. of balloons used					<0.001
1	335/386 (86.8%)	147/191 (77.0%)	123/127 (96.9%)	65/68 (95.6%)	
2	39/386 (10.1%)	32/191 (16.8%)	4/127 (3.1%)	3/68 (4.4%)	
3	10/386 (2.6%)	10/191 (5.2%)	0/127 (0.0%)	0/68 (0.0%)	
4	2/386 (0.5%)	2/191 (1.0%)	0/127 (0.0%)	0/68 (0.0%)	
Diameter [mm]	2.9 (0.5)	3.1 (0.5)	3.0 (0.4)	2.1 (0.3)	<0.001
Length [mm]	19.7 (6.2)	20.6 (6.3)	18.7 (5.9)	18.2 (5.6)	0.003
Pressure [atm]	14.8 (3.3)	15.3 (3.3)	15.0 (3.0)	12.8 (2.9)	<0.001
Inflation time [s]	41.0 (8.9)	41.3 (8.8)	39.1 (7.8)	43.3 (10.6)	0.016
Dissection requiring stenting	10/407 (2.5%)	4/212 (1.9%)	2/127 (1.6%)	4/68 (5.9%)	0.14
Residual stenosis requiring stent	9/407 (2.2%)	5/212 (2.4%)	2/127 (1.6%)	2/68 (2.9%)	0.7
Residual stenosis post-DCB	6.3 ± 16.4	7.6 ± 17.6	3.3 ± 13.4	7.3 ± 16.8	0.002
Residual stenosis > 20%	38/451 (8.4%)	27/249 (10.8%)	6/131 (4.6%)	5/71 (7.0%)	0.102
Perforation	1/386 (0.3%)	1/191 (0.5%)	0/127 (0.0%)	0/68 (0.0%)	0.600
Vascular occlusion	1/386 (0.3%)	1/191 (0.5%)	0/127 (0.0%)	0/68 (0.0%)	0.600
Periprocedural myocardial infarction	5/386 (1.3%)	3/191 (1.6%)	0/127 (0.0%)	2/68 (2.9%)	0.200

**Table 3:** Clinical endpoints at 12 months.

	Total Cohort (n = 386)	DES-ISR (n = 191)	BMS-ISR (n = 127)	De Novo (n = 68)	*p*-Value
ENDPOINTS					
Device success	446/451 (98.9%)	246/249 (98.8%)	129/131 (98.5%)	71/71 (100.0%)	0.8
TLR at 12 months	21/386 (5.4%)	17/191 (8.9%)	3/127 (2.4%)	1/68 (1.5%)	0.013
TVMI at 12 months	8/386 (2.1%)	6/191 (3.2%)	1/127 (0.8%)	1/68 (1.5%)	0.5
Cardiac death at 12 months	7/386 (1.8%)	5/191 (2.6%)	0/127 (0.0%)	2/68 (2.9%)	0.13

## Data Availability

The data presented in this study are available on request from the corresponding author. The data are not publicly available due to institutional policy reasons.

## Supplementary Materials

The following supporting information can be downloaded at: https://www.mdpi.com/article/10.3390/jcm14093133/s1. Supplementary Table S1. Literature for sample size calculation—one year cd-TLR rates for Pantera Lux in DES-ISR; Supplementary Table S2: Literature for sample size calculation—one year cd-TLR rates for Pantera Lux in BMS-ISR; Supplementary Table S3. Literature for sample size calculation—one year cd-TLR rates for comparator DCB in DES-ISR; Supplementary Table S4. Literature for sample size calculation—one year cd-TLR rates for comparator DCB in BMS-ISR; Supplementary Table S5. Literature for sample size calculation—one year cd-TLR rates for Pantera Lux in de-novo lesions; Supplementary Table S6. Literature for sample size calculation—one year cd-TLR rates for comparator DCB in de-novo lesions; Supplementary Table S7. Univariable and multivariable analysis for target lesion revascularization at 12 months.

## Abbreviations

The following abbreviations are used in this manuscript:

DCB	Drug-coated balloon
PCB	Paclitaxel-coated balloon
ISR	In-stent restenosis
DES	Drug-eluting stent
BMS	Bare-metal stent
TLR	Target-lesion revascularization
TV-MI	Target-vessel myocardial infarction
