# Supplementary material for: Pantera Lux Drug-Coated Balloon for the Treatment of Coronary Artery Lesions in Routine Practice
Source: J Clin Med. 2025 May 1;14(9):3133. doi: 10.3390/jcm14093133 (PMC12072451; doi:10.3390/jcm14093133)
Supplement: Supplementary file 1 [file jcm-14-03133-s001.zip › jcm-3534310-supplementary.pdf]

Supplemental Material

Pantera Lux Drug Coated Balloon for the Treatment of Coronary Artery Lesions in Routine Practice

Hemetsberger Rayyan, Mankerious Nader, Hamzaraj Kevin, Alali Ahmad, Richardt Gert, Toelg Ralph

Methods

Short list from the IFU applications for Pantera Lux

- Select an appropriate Pantera Lux catheter for the target vessel. The diameter of the balloon should closely match the reference diameter of the target vessel. The inflation diameter or the balloon must not exceed the diameter of the coronary artery proximal or distal lo the stenosis. Select a balloon length which closely matches the lesion length.
- Gently pull out the catheter from the protection ring without removing the balloon protector in order to avoid any contact with the balloon coating.
- Remove air from the catheter with the balloon protector and transportation wire still in place
- Open the three-way stopcock so that the fluid path between the catheter and the inflation device is established.
- Create a vacuum on the inflation device and remove air from the catheter for at least 30 seconds.
- Close the three-way stopcock so that the fluid path to the catheter is closed and evacuate all air from the inflation device through the stopcock. Repeat if necessary, to ensure that any air contained in the balloon and inflation lumen is removed. Release the inflation barrel to normal pressure.
- Open the three-way stopcock so that the fluid path between the catheter and the inflation device is established.
- Carefully remove the balloon protector together with the transportation wire
- Open the hemostatic valve to an extent that allows inserting the Pantera Lux catheter without any friction. Carefully insert the Pantera Lux catheter through the hemostatic valve.

- Advance the Pantera Lux catheter on the guidewire into the target vessel until it reaches the lesion.
- Depending on patient situation and vessel morphology the inflation should be kept for a period of at least 30 seconds.
- Deflate the balloon in accordance with standard PTCA procedures. Apply negative pressure of the balloon for at least 30 seconds before carefully pulling back the Pantera Lux catheter out of the target vessel.

Sample Size Calculation

The sample size calculation is based on the 12 months cd-TLR rate which is the primary endpoint of the data evaluation, and following a precision-based approach using the exact Clopper-Pearson 95% confidence interval (CI) method. Calculation was performed with PASS 15 Power Analysis and Sample Size Software (2017).

A) Sample size for DES-ISR and BMS-ISR population

Expecting a cd-TLR incidence rate of 9.7% and the corresponding one-sided upper 95% CI being 12.9%, a sample size of 314 patients is required. Assuming missing 1-year post index procedure information in 4 patients, the sample size is 318 patients (191 DES-ISR and 127 BMS-ISR).

The expected incidence rate for Pantera Lux is derived from a cohort consisting of 60% DES-ISR and 40% BMS-ISR (0.6x13.6% + 0.4x3.9%). The expected incidence rate of 13.0% for DCB other than Pantera Lux is derived from a cohort of 60% DES-ISR and 40% BMS-ISR cases (0.6x17.4% + 0.4x6.4%). The chosen precision by means of the one-sided upper 95% CI was defined being below 13.0% and set to 12.9%.

A1) Pantera Lux - DES-ISR:

Four studies reporting cd-TLR rates for Pantera Lux in DES-ISR were identified with rates ranging between 9.8% and 18.9%, and a weighted rate of 13.6% was calculated.

Study name	Patient number, n (Pantera-Lux)	12m cd-TLR rate (%)
PEPPER first-in-man study (1)	38	17.1
DELUX all-comers registry (2)	419	11.5
ISAR-DESIRE 4 (3)	252	18.9
BIOLUX RCT (4)	157	9.8
Weighted rate	-	13.6

Supplemental Table S1. Literature for sample size calculation - one year cd-TLR rates for Pantera Lux in DES-ISR

A2) Pantera Lux - BMS-ISR:

Two studies reporting cd-TLR rates for Pantera Lux in BMS-ISR were identified with rates of 2.4% and 3.6% respectively. A weighted rate of 3.9% was calculated.

Study name	Patient number, n (Pantera-Lux)	12m cd-TLR rate (%)
PEPPER first-in-man study (1)	43	2.4
DELUX all-comers registry (2)	499	4.0
Weighted rate	-	3.9

Supplemental Table S2: Literature for sample size calculation - one year cd-TLR rates for Pantera Lux in BMS-ISR

A3) Comparator DCB – DES-ISR

Four studies reporting cd-TLR rates for comparator DCB in DES-ISR were identified reporting rates between 13.0% and 22.0%. A weighted rate of 17.4% was calculated.

Study name	Patient number, n (Comparator DCB)	12m cd-TLR rate (%)
Multicenter, retrospective observational study (5)	81	19.8
Multicenter, prospective Spanish DIOR registry (6)	61	14.8
RIBS IV (7)	154	13.0
ISAR-DESIRE 3 and ISAR-DESIRE 4 (8)	138	22.0
Weighted rate	-	17.4

Supplemental Table S3. Literature for sample size calculation - one year cd-TLR rates for comparator DCB in DES-ISR

A5) Comparator DCB – BMS-ISR

Four studies reporting cd-TLR rates for comparator DCB in BMS-ISR were identified reporting rates between 4.0% and 9.2%. A weighted rate of 6.4% was calculated.

Study name	Patient number, n (Comparator DCB)	12m cd-TLR rate (%)
Paccocath ISR I / ISR II trial (9)	54	4.0
PEPCAD II trial (10)	66	6.3
Multicenter, prospective Spanish DIOR registry (6)	65	9.2
RIBS IV (7)	95	6.0
Weighted rate	-	6.4

Supplemental Table S2. Literature for sample size calculation - one year cd-TLR rates for comparator DCB in BMS-ISR

B) Sample size for de-novo lesions

Expecting a cd-TLR incidence rate of 2.9% and the corresponding one-sided 95% CI being 9.1%, a sample size of 66 patients is required. Accounting for the potential loss of 1-year post-procedure data in 2 patients, the total sample size is adjusted to 68 patients.

The expected incidence rate for Pantera Lux is derived from two publications. The expected incidence rate of 9.2% for DCB other than Pantera Lux is derived from three publications. The chosen precision by means of the one-sided upper 95% CI was defined being below 9.2% and set to 9.1%.

B1) Pantera Lux – *de-novo* lesions

Two studies reporting 12-month cd-TLR rates for Pantera Lux in *denovo* lesions were identified with rates of 2.9% and 3.1% respectively. A weighted rate of 2.9% was calculated.

Study name	Patient number, n (Pantera-Lux)	12m cd-TLR rate (%)
DELUX all-comers registry (2)	111	3.1
PANELUX (11)	432	2.9
Weighted rate	-	2.9

Supplemental Table S5. Literature for sample size calculation - one year cd-TLR rates for Pantera Lux in de-novo lesions

B2) Comparator DCB – *de-novo* lesions

Three studies reporting cd-TLR rates for comparator DCB in *de-novo* lesions were identified with rates between 5.3% to 11.9%. A weighted rate of 9.2% was calculated.

Study name	Patient number, n (Comparator DCB)	12m cd-TLR rate (%)
PEPCAD I (12)	118	11.9
BELLO (diabetic pts) (13)	39	5.3
BELLO (non-diabetic pts) (13)	51	5.9
Weighted rate	-	9.2

Supplemental Table S3. Literature for sample size calculation - one year cd-TLR rates for comparator DCB in de-novo lesions

Results

	Univariable		Multivariable	
**TLR**	OR [CI 95%]	p-value	OR [CI 95%]	p-value
**DES-ISR**	4.52 [1.62;16.4]	0.003	4.67 [1.73–15.46]	0.002
**BMS-ISR**	0.34 [0.07-1.03]	0.058		
**De-novo**	0.25 [0.01-1.24]	0.103		
**Age per year**	0.98 [0.94-1.02]	0.326		
**Female**	0.8 [0.31-1.97]	0.637		
**BMI**	1.01 [0.91-1.11]	0.862		
**Hyperlipidemia**	2.51 [0.90-9.09]	0.082		
**Hypertension**	2.68 [0.35-345]	0.425		
**Diabetes mellitus**	1.35 [0.51-3.32]	0.531		
**Smoking history**	0.85 [0.32-2.09]	0.726		
**LVEF per 1%**	0.97 [0.94-1.01]	0.095		
**Previous PCI**	1.14 [0.22-28.0]	0.905		
**Peripheral artery disease**	1.68 [0.52-4.54]	0.354		
**Carotid artery disease**	0.61 [0.13-1.88]	0.418		
**Impaired renal function**	1.91 [0.65-4.94]	0.226		
**Clinical presentation**				
**STEMI**	9.67 [1.14-56.1]	0.040	43.10 [2.67–6,608.15]	0.007
**NSTEMI**	4.84 [1.59-13.1]	0.007	16.53 [1.75–2,212.41]	0.010
**Unstable angina**	0.30 [0.002-2.27]	0.308		
**Chronic coronary syndrome**	0.31 [0.12-0.81]	0.019	3.09 [0.38–401.23]	0.359

Supplemental Table S7. Univariable and multivariable analysis for target lesion revascularization at 12 months

References

1. Hehrlein C, Dietz U, Kubica J, Jorgensen E, Hoffmann E, Naber C, et al. Twelve-month results of a paclitaxel releasing balloon in patients presenting with in-stent restenosis First-in-Man (PEPPER) trial. Cardiovasc Revasc Med. 2012;13(5):260-4.
2. Toelg R, Merkely B, Erglis A, Hoffman S, Bruno H, Kornowski R, et al. Coronary artery treatment with paclitaxel-coated balloon using a BTHC excipient: clinical results of the international real-world DELUX registry. EuroIntervention. 2014;10(5):591-9.
3. Kufner S, Joner M, Schneider S, Tolg R, Zrenner B, Repp J, et al. Neointimal Modification With Scoring Balloon and Efficacy of Drug-Coated Balloon Therapy in Patients With Restenosis in Drug-Eluting Coronary Stents: A Randomized Controlled Trial. JACC Cardiovasc Interv. 2017;10(13):1332-40.
4. Jensen CJ, Richardt G, Tolg R, Erglis A, Skurk C, Jung W, et al. Angiographic and clinical performance of a paclitaxel-coated balloon compared to a second-generation sirolimus-eluting stent in patients with in-stent restenosis: the BIOLUX randomised controlled trial. EuroIntervention. 2018;14(10):1096-103.
5. Basavarajaiah S, Naganuma T, Latib A, Sticchi A, Ciconte G, Panoulas V, et al. Treatment of drug-eluting stent restenosis: Comparison between drug-eluting balloon versus second-generation drug-eluting stents from a retrospective observational study. Catheter Cardiovasc Interv. 2016;88(4):522-8.
6. Vaquerizo B, Serra A, Miranda-Guardiola F, Martinez V, Antoni Gomez-Hospital J, Iniguez A, et al. One-year outcomes with angiographic follow-up of paclitaxel-eluting balloon for the treatment of in-stent restenosis: insights from Spanish multicenter registry. J Interv Cardiol. 2011;24(6):518-28.
7. Alfonso F, Perez-Vizcayno MJ, Cardenas A, Garcia del Blanco B, Garcia-Touchard A, Lopez-Minguez JR, et al. A Prospective Randomized Trial of Drug-Eluting Balloons Versus Everolimus-Eluting Stents in Patients With In-Stent Restenosis of Drug-Eluting Stents: The RIBS IV Randomized Clinical Trial. J Am Coll Cardiol. 2015;66(1):23-33.
8. Colleran R, Joner M, Kufner S, Altevogt F, Neumann FJ, Abdel-Wahab M, et al. Comparative efficacy of two paclitaxel-coated balloons with different excipient coatings in patients with coronary in-stent restenosis: A pooled analysis of the Intracoronary Stenting and Angiographic Results: Optimizing Treatment of Drug Eluting Stent In-Stent Restenosis 3 and 4 (ISAR-DESIRE 3 and ISAR-DESIRE 4) trials. Int J Cardiol. 2018;252:57-62.

9. Scheller B, Hehrlein C, Bocksch W, Rutsch W, Haghi D, Dietz U, et al. Two year follow-up after treatment of coronary in-stent restenosis with a paclitaxel-coated balloon catheter. Clin Res Cardiol. 2008;97(10):773-81.
10. Unverdorben M, Vallbracht C, Cremers B, Heuer H, Hengstenberg C, Maikowski C, et al. Paclitaxel-coated balloon catheter versus paclitaxel-coated stent for the treatment of coronary in-stent restenosis. Circulation. 2009;119(23):2986-94.
11. Roncalli J, Godin M, Boughalem K, Shayne J, Piot C, Huret B, et al. Paclitaxel Drug-Coated Balloon After Bare-Metal Stent Implantation, an Alternative Treatment to Drug-Eluting Stent in High Bleeding Risk Patients (The Panelux Trial). J Invasive Cardiol. 2019;31(4):94-100.
12. Unverdorben M, Kleber FX, Heuer H, Figulla HR, Vallbracht C, Leschke M, et al. Treatment of small coronary arteries with a paclitaxel-coated balloon catheter. Clin Res Cardiol. 2010;99(3):165-74.
13. Giannini F, Latib A, Jabbour RJ, Costopoulos C, Chieffo A, Carlino M, et al. Comparison of paclitaxel drug-eluting balloon and paclitaxel-eluting stent in small coronary vessels in diabetic and nondiabetic patients - results from the BELLO (balloon elution and late loss optimization) trial. Cardiovasc Revasc Med. 2017;18(1):4-9.
